# Glycaemic Index and Load Values Tested in Normoglycemic Adults for Five Staple Foodstuffs: Pounded Yam, Pounded Cassava-Plantain, Placali, Attieke and Maize Meal Stiff Porridge

**DOI:** 10.3390/nu7021267

**Published:** 2015-02-16

**Authors:** Adam C. Kouamé, Kouakou N. Kouassi, Yao D. N’dri, N’guessan G. Amani

**Affiliations:** 1 Laboratory of Food Biochemistry and Tropical Products Technology, Nutrition Section, Department of Food Science and Technology, Nangui Abrogoua University, PO Box 801 Abidjan 02, Cote D’Ivoire; E-Mail: nestorkksi@yahoo.fr; 2 Laboratory of Food Biochemistry and Tropical Products Technology, Biochemistry and Food Technology Section, Department of Food Science and Technology, Nangui Abrogoua University, PO Box 801 Abidjan 02, Cote D’Ivoire; E-Mails: ndri_denis@yahoo.fr (Y.D.N.); amanigeorges@yahoo.fr (N.G.A.)

**Keywords:** glycaemic index, glycaemic load, normoglycaemic adults, staple foodstuffs, gelatinisation, attieke agbodjama, Côte d’Ivoire

## Abstract

There is currently an increased global interest in the published glycaemic index (GI) and glycaemic load (GL) values of foods. However, data on the GI and GL values of different varieties of foods within Côte d’Ivoire are very limited. The study therefore aimed at finding the GI and GL of the main food staples in Côte d’Ivoire. Following the International Standard Organisation’s protocol (ISO/FDI 26642:2010), a selection of five staple foodstuffs were tested for their GI and GL. Fasted healthy subjects were given 50 g of available carbohydrate servings of a glucose reference, which was tested twice, and test foods which were tested once, on separate occasions. Excepted attieke (GI 63), the majority of foods tested have a high GI (GI > 70). Attieke (agbodjama) had a high GL (GL 29) while placali (GL 17) and maize meal stiff porridge (GL 16) had medium GLs. The GLs of pounded cassava-plantain and pounded yam are 26 and 22. Consumption of attieke could minimize postprandial blood glucose spikes, in spite of high GL and potentially have benefit in the management and prevention of some chronic diseases.

## 1. Introduction

Roots, tubers, plantains and cereals are the traditional staples in many countries and play important roles in the diet of populations around the world [1]. They are not only rich in starch and the major source of energy, but also contain vitamins, minerals, phytoestrogens, and trace elements [2]. Yam (tubers), plantain (fruits), cassava (roots) and maize (cereals) are considered as the main carbohydrate sources in Ivorian diets [3]. These staple crops are used in preparing typical Ivorian dishes such as: foutou (from yam, cassava, plantain or mixed cassava-plantain), cabatôh or tôh de maïs (maize meal stiff porridge), attieke and placali (from cassava) and aloco (from ripe plantain) [4]. Generally these foods are eaten with various vegetables and other food complements sauces [5]. There is some concern over their consumption, as some are of the view that such traditional starch-based diets could be detrimental to health, since the regular consumption of high starch contents may trigger hyper-postprandial glucose responses. Some people, especially diabetic patients, even exclude grains or tubers from their diets. This is largely because of a lack of knowledge of the glycaemic index (GI) and glycaemic load (GL) values for many staple foods in Côte d’Ivoire. The determination of the glycaemic responses of these foods therefore is required considering its role in dietary management sugar related disease conditions (*i.e.*, to maintain normoglycaemia and possibly also maintain insulin demand). The five most available starchy foods that are controversial regarding to their glycaemic effects on humans were chosen for this study. The practical use of GI/GL of foods may support the establishment of optimum dietary recommendations and good eating habits.

## 2. Experimental Section

### 2.1. Setting

The study was conducted at the Department of Foods Sciences and Technologies of University Nangui Abrogoua using internationally recognized GI methodology [6,7]. All clinical procedures were carried out at the Endocrinology and Diabetology Center, CHU Yopougon, Abidjan, Côte d’Ivoire.

### 2.2. Subjects

Subjects were identified from student and staff populations of Nangui Abrogoua University. Each food item was consumed by 10 subjects to provide the statistical power required for the data analysis. Subjects were grouped accordingly. Interested subjects were asked to complete a health-screening questionnaire to check against ill health, including clinically abnormal glucose metabolism (fasting blood glucose < 6.0 mmol/L) and any medical conditions or medications that might affect glucose regulation, gastric emptying, body weight, appetite and energy expenditure. Demographic profiles of age, gender and anthropometric measurements were carried out for all the subjects using standardized methods before the study began. Height was recorded to the nearest centimeter using a Stadiometer (Seca Limited, Birmingham, West Midlands, UK) with the subjects standing erect without shoes. Body weight was recorded using the Tanita BC-418 MA (Tanita UK Limited, Yiewsley, Middlesex, UK) with the subjects wearing light clothing and no shoes. Body mass index (BMI) was calculated by dividing body weight (kg) by the square of the height (m^2^). Blood pressure was measured with an automatic device (A & D Company Ltd., Tokyo, Japan) in subjects. Mean arterial blood pressure (BP) was calculated as one third of the systolic plus two thirds of the diastolic BP. For indication of blood glucose control, glycated haemoglobin was analyzed, and subjects with an HbA1c <8% were included in the study. Determination of HbA1c was achieved through the DiaStat for measuring HbA1c reported to the total HbA. Inclusion/exclusion criteria: only non-diabetic individuals between the ages of 25 and 45 years, with BMI between 18.5 and 25.0 kg/m^2^ and fasting blood glucose value lower than 6.4 mmol/L were eligible to participate in the study. Smokers, overweight, and obese individuals were excluded from the study. Emphasis was placed on subjects who were healthy, with an active lifestyle, without any diagnosed diseases. During the study, subjects were advised to continue their customary daily activities without any change in their physical activities.

### 2.3. Study Protocol

The study was carried out using an international standard GI test protocol (ISO/FDIS 26642:2010 Food products—Determination of the glycaemic index (GI) and recommendation for food classification) as outlined by Finocchiaro *et al.* [8] and is in line with procedures recommended by the Food and Agricultural Organization of the United Nations/World Health Organization [6]. Glucose (50 grams of Glucose pur anhydre, COOPER, Place Lucien Anver, dissolved in 250 mL water) was used twice as the reference food. In the current study, each product was tested on ten subjects. On the day before a test, subjects were asked to restrict their intake of alcohol and caffeine-containing drinks and to restrict their participation in intense physical activity. Subjects were also told not to eat or drink after 21:00 h the night before a test, although water was allowed in moderation.

### 2.4. Test Foods Procedure—Collection of Samples and Description/Preparation of Experimental Diets

A number of different foods were tested, including the traditional foods prepared from yam, cassava, plantain and maize. These raw materials represent a diverse range of foods commonly consumed in Côte d’Ivoire. They were selected according to the frequency of their consumption, their estimated content in one or several micronutrients of interest (zinc, iron, vitamin A), or the quantity ingested per meal [3,9]. The ingredients and raw material used for this study were purchased from Market of Port-Bouët in District of Abidjan (Côte d’Ivoire). From raw materials, five traditional dishes usually consumed by the Ivorian population were tested: pounded yam (foutou d’igname), pounded plantain (foutou de banane), cassava paste fermented (placali), fermented cassava couscous (attieke or agbodjama) and maize meal stiff porridge or maize porridge (cabatôh). The foods were prepared and cooked in the traditional manner by the staff of a university-based cafeteria which had been given specific and detailed preparation instructions (Table 1).

In accordance with the guidelines in the document ISO 2010 (ISO/FDIS 26642:2010 Food products—Determination of the glycaemic index (GI) and recommendation for food classification), subjects tested each test food once and the reference food twice in random order on separate days, with at least a 1 day gap between measurements to minimize carry-over effects. Subjects were studied in the morning after a 12 h overnight fast. Subjects consumed the reference or test foods at a comfortable place, within 15 min. The test foods and reference food were served with 250 mL water. A further 250 mL water were given during the subsequent 2 h. Subjects were encouraged to keep physical activity to a minimum during the testing.

**Table 1:** Processing and preparation of foods.

Food (Raw Material Used) Agricultural Form	Test Food Description/Preparation
White yam tuber based (*Dioscorea cayenensis*-rotundata; variety *Kponan*)	Group 1: Pounded yam or yam foutou. Yam has taken the first place in rank with its volume (6,932,950 tons per year), among the consumer crops produced in Côte d’Ivoire [3]. Generally, *Kponan* variety is mainly used for the preparation of foutou [10]. For pounded yam production, the tubers were peeled, cut into pieces and boiled until soft. The water is then drained off and the pieces pounded in a wooden mortar and pestle until stiff glutinous dough is formed, usually taking 15–30 min [11,12].
Cassava root based food items (*Manihot esculenta Crantz*; Improved African Cassava (IAC) variety)	Group 2: Attieke (a fermented cassava couscous). The most popular food derived from fermented cassava is attieke [13]. This product is widely consumed in Côte d’Ivoire and in neighbouring countries [14]. It is a traditional foodstuff made by fermentation and steam-cooking cassava root [15]. In Côte d’Ivoire, attieke is mainly prepared from the local variety called Improved African Cassava (IAC), which is bitter, pest-resistant and produces high yields [16,17]. Attieke processing technology comprises peeling the roots, reducing them into mash, inoculating obtained dough with a cooked and fermented cassava pulp and adding palm oil. The fresh mash is fermented for two or three days, mechanically squeezed in order to remove as much water as possible, granulated, sun-dried before sieving and finally steamed to get the final product attieke [14,15].
	Group 3: Placali (a fermented cassava paste). Bitter cassava roots are usually used to prepare placali. In Côte d’Ivoire, placali is commonly consumed and it is the second well-known cassava product after attieke [18]. The placali meal was prepared as follows: cassava are peeled, crushed and mixed with a small amount of fermented cassava. The paste obtained is fermented for one to two days and then sieved to remove fibers. The fermented dough is transformed into a gel called “placali” after simmering [19].
Plantain based (*Musa paradisiaca* L.; variety *ameletia*) *Musa* spp., AAB group, cv. False Horn in ripeness stage 5.	Group 4: Pounded plantain or pounded cassava-plantain (pounded cassava mixed with pounded plantain). The plantain and cassava are washed, peeled and plantain is sliced lengthways into two with a stainless steel knife to cut out the black spots (atrophied seeds). Peeled cassava (750 g) and ripe plantain pieces (2 kg) are cooked in water for 1 h. After cooling, the water was drained and the boiled plantain/cassava was transferred into a traditional wooden mortar and pounded to obtain a smooth consistent paste. Pounded plantain sample was moulded into spherical balls [20].
Maize based (*Zea mays* L.)	Group 5: Maize meal stiff porridge or cabatôh. This is a traditional Ivorian recipe from a classic starchy staple made from corn dough boiled in water until it forms a stiff porridge-like paste. It is also known as cabatôh or tôh de mais and is made from cornmeal. The maize meal was prepared as follows: flour of maize (1.2 kg) obtained by pounding whole grains in a mortar, is poured into boiling water (3 L) and stirred until a solid paste is formed.

### 2.5. Physico-Chemical Analyses

The proximate compositions for the foods were determined using a standard Association of Official Analytical Chemists [21] method and the available carbohydrate content for each test meal calculated by difference using the FAO/WHO procedure [6]. All determinations reported were carried out in triplicate. Moisture content determined by drying at 105 °C with constant weight, the protein content determined by the method of Kjeldahl with 6.25 as conversion factor, the lipid content determined by Soxhlet extraction with ether, and the ash content determined by incineration at 650 °C in a muffle furnace; the total dietary fiber content determined by the method of Prosky [22], the carbohydrate content determined by difference. The energy content of the sample was computed from the proximate data using the Atwater formula [23]. Table 1 lists the composition and preparation of the test foods. All foods were tested immediately after cooking. Foods were bought and prepared on the day of testing following the common practices in Côte d’Ivoire. All foods were tested in equivalent available carbohydrate amounts (50 g) and compared with a reference food (50 of glucose). Available carbohydrate was provided following to the nutrition information available from the chemical analysis.

### 2.6. Blood Glucose Measurements

Fasting capillary blood samples were taken at 0 min and test foods were consumed immediately. Further blood samples were taken at 15, 30, 45, 60, 90 and 120 min after starting to eat. Blood was obtained by finger-prick using the Accu-Chek^®^ Fastclix Lancing Device (Castle Hill, NSW, Australia). Before a finger-prick, subjects were encouraged to warm their hand to increase blood flow. Fingers were not squeezed to extract blood from the fingertip in order to minimize plasma dilution. Blood glucose was measured using a calibrated Accu-Chek^®^ Performa glucometer (Accu-Chek Performa, Roche Diagnostic, Castle Hill, NSW, Australia). This procedure was done in strict compliance with the protocol recommendations of the manufacturer as well as good laboratory practice as described in the ISO guidelines (ISO/FDIS 26642:2010 Food products—Determination of the glycaemic index (GI) and recommendation for food classification).

### 2.7. Incremental Area under the Curve, Glycaemic Index and Glycaemic Load

The blood glucose values for every point in time over 2 h were used to calculate the incremental area under the curve (iAUC) for each subject and each test individually. The incremental area under the blood glucose response curves to test and reference foods were calculated geometrically using the trapezoid rule, ignoring the area beneath the baseline. The iAUC for each test food eaten by each subject was expressed as a percentage of the mean iAUC glucose for the two repeats of the isocarbohydrate reference food (glucose) consumed by the same subject:

GI = (iAUC test food/iAUC reference food) × 100. (1)

The GI of each food was then calculated as the mean value across all subjects consuming that food. In the case, the individual GI values for any subject were greater or less than 2 SD of the group mean, the GI will be considered as outliers and were excluded from the analysis. The mean, standard deviation and coefficient of variation (CV) of the iAUC of each subject’s repeated reference food were calculated. The glycaemic load (GL) of a typical serving of each food was calculated using the formula below [24]:

GL = (GI × grams of carbohydrate in the typical serving size/100). (2)

### 2.8. Ethical Considerations

This study was conducted according to the guidelines laid down in the Declaration of Helsinki and the University Research, and the Ethics Committee at Félix Houphouet Boigny University of Cocody approved on 12 December 2009 all procedures involving human subjects. The subjects were made aware of the study objectives and of what was expected from them. Participants were given complete details of the study protocol and were given the opportunity to ask questions. They were informed that participation was voluntary, that they were free to withdraw at any time if they no longer wished to participate, and that confidentiality pertaining to the data that were obtained from them would be protected. The participants provided written consent before participating. Each recruited subject was assigned an identity number that was used throughout the test period, as well as in the data entry and analysis. Subjects received a financial reward for their participation.

### 2.9. Data Management

Data were entered in a form designed for the study. Research assistants checked the case report forms for completeness and accuracy. Forms that had been checked and found to be complete and accurate were then signed for purposes of quality control. In preparation for the analysis, data were entered into a Microsoft Excel^®^ spreadsheet. Password restrictions on the use of the project work stations were employed to ensure security and confidentiality of the data.

### 2.10. Statistical Analysis

Data were analyzed according to the method recommended by the norm ISO/FDIS 26642:2010 for the foods product. iAUC and GI/GL were calculated using Microsoft Excel^®^ 2013. Data are presented as means, standard deviations (SD) and range. Comparisons between the foods were made by using one-way analysis of variance (ANOVA) and the Tukey’s multiple comparisons test. Levels of inter- and intra-individual variation of the standard (glucose) tests were assessed by determining the CV [CV% = 100 × (SD/mean)]. Spearman’s correlation coefficient (ρ) was used to assess the relationship between GI values and nutrient content of the test foods. Statistical significance was set at *p* < 0.05. All Statistical analysis was performed using SPSS^®^ 17.0 Statistical program (Statistical Package for Social Sciences, Inc., Chicago, IL, USA).

## 3. Results

### 3.1. Descriptive Characteristics of Subjects

Eighty-two individuals responded to the call for volunteers to participate in the study. All of them were Nangui Abrogoua University students, representing different faculties and levels of education. Participants completed questionnaires regarding age, date of birth, weight, height, carbohydrate metabolism deficiencies, smoking habits, carbohydrate source, physical activity, and medical history. Fifty-nine met the stipulated requirements. Fifty normoglycaemic healthy volunteers completed all parts of the study. This sample consisted of 23 women and 27 men, aged 28 years (SD 3), with a mean body mass index (BMI) (SD) of 21.5 kg/m^2^ (SD 1.2), systolic blood pressure of 107.4 mmHg (SD 8.5), diastolic blood pressure of 72.0 mmHg (SD 7.3) and a fasting blood glucose of 4.6 mmol/L (SD 0.4). Mean HDL (SD) was 0.4 mmol/L (SD 0.1).

### 3.2. Chemical Composition

The proximate composition of analyzed foods is shown in Table 2. Some significant (*p* < 0.05) differences were observed in the proximate composition of foods. The available carbohydrate content of the foods with the serving sizes containing the 50 g available carbohydrate portions are shown in Table 3. The serving sizes were calculated to be 197.5 g of pounded yam, 317.1 g of placali, 173.9 g of pounded plantain, and 231.5 g of maize meal stiff porridge and 108.2 g of attieke.

### 3.3. Within-Subject Variation of Reference Food

The mean intra-individual CV of glycaemic responses (GR)s to the two 50 g glucose standard tests for the subjects of each food group tested was lower than 15%. The inter-individual CV in GR to the standard tests was lower than 25%. These values are consistent because low mean within-subject variation (reference CV < 30%) is required for accuracy [7].

### 3.4. Blood Glucose Response to Test Meals

**Figure 1:**
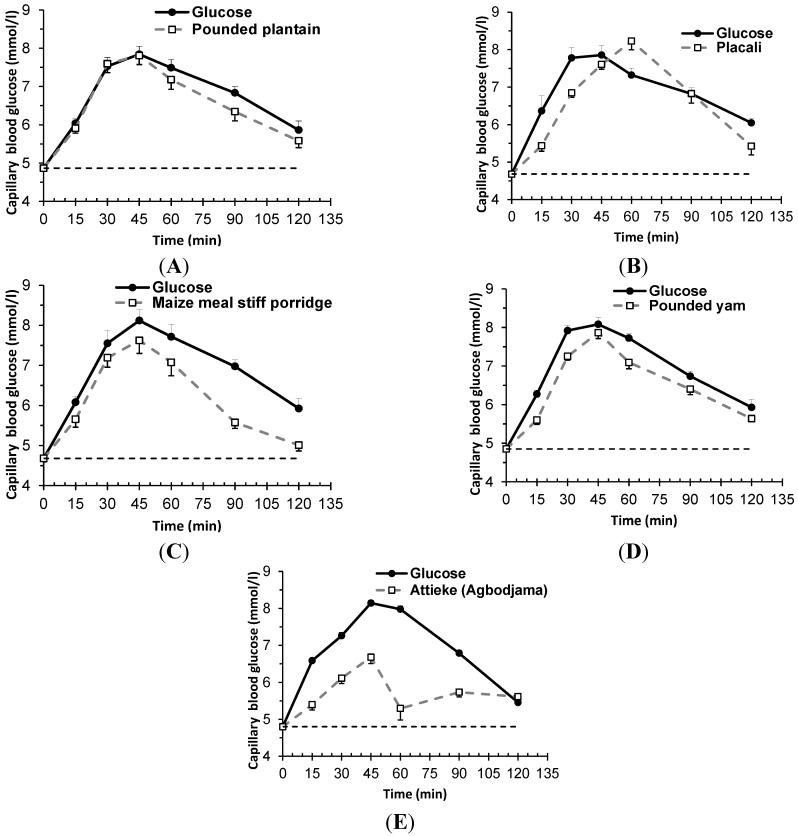
Graphs showing mean blood glucose concentrations for five traditional food product of Côte d’Ivoire (□ (**A**) Pounded plantain (pounded cassava-plantain), (**B**) placali, (**C**) maize meal stiff porridge (cabatôh), (**D**) pounded yam (foutou d’igname), (**E**) attieke); ● glucose). Values are the mean change in blood glucose (BG) with their standard deviation represented by vertical bars (*n* = 10 persons per meal).

The glycaemic profiles for the five meals tested are graphically presented in Figure 1. Blood glucose response curves were intermediate for attieke (E) and high for the other foods (placali (B), pounded yam (D), pounded cassava-plantain (A) and maize meal stiff porridge (C)).

### 3.5. Glycaemic Index/Load Testing Portions

The mean iAUC (mmol × min/L), GI and GL values for all tested foods are given in Table 3. The GI values of the foods tested ranged from 63 (attieke) to 106 (placali). For practical application, GI values are often grouped into categories of low, medium or high GR: low (<55); medium (56–69 inclusive); high (>70) [7]. Thus, most of the test foods fell into the high GI category except attieke which fell into the medium GI category. Also, GL values were classified as low (≤10), medium (>10 to <20) or high (≥20) [25].

### 3.6. Factors that Affect the GI of Tested Foods

There are several factors (*i.e.*, moisture: Spearman’s ρ = 0.268; total dietary fiber: Spearman’s ρ = 0.407; lipids: Spearman’s ρ = −0.469 or energy: Spearman’s ρ = −0.352; *p* < 0.01) that may alter the GI of foods tested. However, in the present study, there was no relationship between the GI value and the amount of protein per 50 g available carbohydrate portion (Spearman’s ρ = 0.01; *p* = 0.89). This was expected, as previous findings from Wolever *et al.* [26] and Henry *et al.* [27] also demonstrate that the amount of protein found in commonly consumed foods does not significantly affect the GR.

**Table 2:** Proximate composition of selected Ivorian starchy foodstuffs (g/100 g wet sample).

Food Samples	Moisture Content (g/100 g)		Dry Matter Content (g/100 g)		Ashes Content (g/100 g)		Total Dietary Fiber Content (g/100 g)		Proteins Content (g/100 g)		Lipids Content (g/100 g)		Available CHO Content (g/100 g) *		Total Content CHO (g/100 g) *		Energetic Value (kcal/100 g) **	
	Mean (range)	SD	Mean (range)	SD	Mean (range)	SD	Mean (range)	SD	Mean (range)	SD	Mean (range)	SD	Mean (range)	SD	Mean (range)	SD	Mean (range)	SD
Pounded yam	71.0 ^c^ (70.9–71.0)	0.0	29.0 ^d^ (29.0–29.1)	0.0	0.8 ^b^ (0.8–0.8)	0.0	0.6 ^b^ (0.6–0.7)	0.2	1.5 ^b^ (1.5–1.6)	0.0	0.8 ^b^ (0.8–0.8)	0.0	25.3 ^c^ (25.3–25.4)	0.1	25.9 ^d^ (25.9–26.0)	0.0	114.3 ^c^ (114.0–114.8)	0.4
Placali	81.0 ^a^ (81.0–81.0)	0.0	19.0 ^f^ (19.0–19.0)	0.0	0.8 ^b^ (0.8–0.8)	0.0	1.6 ^a^ (1.5–1.7)	0.1	0.8 ^c^ (0.8–0.9)	0.0	0.0 ^e^ (0.0–0.0)	0.0	15.8 ^d^ (15.7–15.8)	0.1	17.4 ^c^ (17.3–17.4)	0.0	66.5 ^d^ (66.7–66.9)	0.5
Pounded cassava-plantain	68.3 ^d^ (68.2–68.6)	0.2	31.7 ^c^ (31.4–31.8)	0.2	0.2 ^c^ (0.2–0.3)	0.0	0.6 ^b^ (0.6–0.6)	0.0	1.7 ^b^ (1.6–1.7)	0.0	0.4 ^c^ (0.4–0.4)	0.0	28.8 ^b^ (28.5–28.9)	0.2	29.3 ^b^ (29.1–29.5)	0.2	125.4 ^b^(124.5–126.1)	0.3
Maize meal stiff porridge	73.4 ^b^ (73.0–73.7)	0.4	26.6 ^e^ (26.3–27.0)	0.4	1.1 ^a^ (1.0–1.3)	0.1	1.5 ^a^ (1.4–1.6)	0.1	2.2 ^a^ (2.1–2.3)	0.1	0.2 ^d^ (0.2–0.2)	0.0	21.6 ^e^ (21.3–22.0)	0.4	23.1 ^e^ (22.7–23.6)	0.5	97.0 ^e^ (96.2–98.4)	1.2
Attieke	51.2 ^e^ (49.3–52.3)	1.7	48.8 ^b^ (47.8–50.7)	1.7	0.7 ^b^ (0.7–0.7)	0.0	0.2 ^c^ (0.2–0.2)	0.0	0.4 ^d^ (0.4–0.4)	0.0	1.3 ^a^ (1.3–1.3)	0.0	46.2 ^a^ (45.1–48.1)	1.7	46.4 ^a^ (45.3–48.3)	1.7	198.1 ^a^ (193.9–205.7)	6.6

Data are means and standard deviation (SD) of three trials; ^a, b, c, d, e, f^ Data on the same column with different letter superscripts are significantly different (*p* < 0.05) as assessed by Tukey’s test; * Calculated by difference of moisture content, ash, fiber, lipids and protein; ** Energy Value = (4 × % Available carbohydrates) + (4 × % Protein) + (9 × % Fat) (kcal/100g).

**Table 3:** Incremental area under the curve and glycaemic index/load values of five staple foods.

Food	Available CHO ^†^ (g/100 g of Food)	Experimental Portion (g)	GI ^1^ (Glucose = 100) ^3^			GL ^2^ (per Experimental Portion Size)		
			Mean	SE	Category	Mean	SE	Category
Pounded yam	25.3	197.5	85 ^b^	4	high	22 ^cd^	1	high
Placali	15.8	317.1	106 ^a^	5	high	17 ^de^	1	medium
Pounded plantain	28.8	173.9	91 ^ab^	4	high	26 ^bc^	1	high
Maize meal stiff porridge	21.6	231.5	74 ^bc^	5	high	16 ^e^	1	medium
Attieke	46.2	108.2	63 ^c^	2	medium	29 ^b^	1	high

^a, b, c, d, e^ Mean values within a column with unlike superscript letters are significantly different (*p* < 0.05) as assessed by Tukey’s test; **^†^** Calculated according to FAO/WHO [6]. Values are Mean and Standard Error (SE); GI, glycaemic index; GL, glycaemic load; CHO, carbohydrate; *n* = 10 persons per meal; ^1^ Level of glycaemic indexes (GIs) were classified according to high (>69), medium (56–69 inclusive) and low (<56) GI; ^2^ level of glycaemic loads (GLs) were classified as high (≥20), medium (>10 to <20), and low (≤10) GL; ^3^ glucose was used as reference food and was defined as 100.

## 4. Discussion

The aim of this study was to determine the GI/GL of a selection of five popular foodstuffs frequently consumed in the Ivorian diet and to consider some factors that influence the GI of these foods. The GIs of foods described in Table 1 were determined using a standardised [7] and quite robust protocol. Of the five staple foodstuffs tested in normoglycaemic adults, attieke agbodjama recorded the lowest GI value (GI 63 (SD 7)). The other four foods had a wide range of GI values (74–106): maize meal stiff porridge (GI 74 (SD 16)), pounded yam (GI 85 (SD 14)), pounded cassava-plantain (GI 91 (SD 11)) and placali (GI 106 (SD 17)) (Table 3). The majority of GI values of foods tested (with the exception of attieke) have been identified as high GI [7]. Similar foods like the amala (yam tuber: *Dioscorea rotundata*), agidi (maize: *Zea mays*) and eba/garri (cassava tuber: *Manihot utilisima*) studied by Omoregie and Osagie [28] in Nigeria had GI ranging from 82 to 99. Mahgoub *et al.* [29] in Botswana also found GIs of the key staple carbohydrate rich foods prepared from wheat (GI 103), maize (GI 91), sorghum (GI 92), millet (GI 95) and legumes (GI 86). Due to their high carbohydrate contents (80% dry weight) and the presence of gelatinised starch (gelatinisation is determined by free moisture), these foods have been capable of inducing significant glycaemia in normal serving sizes, contrary to attieke, which could be considered as a medium GI food (GI 55–69) [7]. High GI foods are known to produce high GR as a result of the fast rate of digestion of carbohydrate in the intestinal lumen hence, the high absorption of glucose into circulation [30]. These foods are not suitable or adequate meals for type II diabetics considering their GI classification [7]. For the preparation of recipes, different processing technologies were used to produce the raw materials, such as pounding for pounded yam and pounded cassava-plantain, crushing and fermentation for placali, attieke and maize meal stiff porridge or the addition of palm oil for attieke. Cooking methods such as ebullition for the placali, pounding for cassava-plantain and pounded yam meals and the steam cooking for attieke, were employed (Table 1). These processing methods induce gelatinisation, thereby permanently disrupting the amylose/amylopectin structure of the starch complex and making it more accessible to digestive enzymes. The method of processing of an individual food can greatly change its GI [31]. Examples of such foods include attieke (GI 63) and placali (GI 106), which are both made of cassava. Indeed, in the preparation of fermented cassava paste (placali) in boiled water, the high temperature and humidity modifies the physical and granular structure of starch and therefore increases its digestibility and impact on blood glucose level. Starch granules take up water and swell, which irreversibly disrupts the crystalline structure of the starch, making it able to be readily hydrolysed by amylase. These gelatinized starch molecules are more easily digested because of particle disintegration [32,33]. With respect to attieke, its relatively low GI could be due to the mechanical squeeze (in order to remove as much as water possible), granulation and sun-drying process. The relative low temperature of the drying process leads to a phenomenon called retrogradation, which changes its conformation and makes it more resistant to digestion [34]. Therefore, the starch stays partially gelatinized in an important way under a few more shapes resistant to an ulterior hydration (steamed-cooking). In addition, during the granulation process, the large particle size of semoula makes the starch gelatinisation relatively difficult and thus slows the enzyme attack. This results in slow release of glucose from food and prevents the high peaks in postprandial blood glucose levels. Of course, the degree of gelatinisation of foods tested has not been measured in the present study, but the low moisture in attieke (52.2 g/100 g) *vs.* 80.0 g/100 g for placali (*p* = 0.001, Table 2) can easily demonstrate its low gelatinisation and above all that gelatinisation is determined by free moisture [35]. An increase in the cooking temperature has a positive effect on gelatinisation and is greater as moisture increases [35,36].

Food factors that can influence the GI include processing, preparation and cooking methods, the physical form of the food, type of sugars and starch in the food, presence of other macronutrients and anti-nutrients, and the ripeness or maturity of the food [37]. However in the present study, only the effect of protein content was not correlated with GI of foods according to the correlation analysis. This is consistent with previous findings of Wolever *et al.* [26] and Henry *et al.* [27] who demonstrated that a small amount of protein in foods, as observed in this study (4.3–8.3 g/100 g dry weight), does not significantly affect the GR. At least 20–30 g dietary protein is needed to increase insulin responses sufficiently to reduce GRs [38,39]. In addition, the high lipid content of attieke (2.6 g/100 g; *p* < 0.05) compared to the other products, could have influenced the digestion of attieke and resulted in the relative low GI, as determined by the negative correlation (Spearman’s ρ = −0.469; *p* < 0.01) between the GI values and lipid intake. Even though the lipid contents in attieke and pounded yam are similar (lipids rate: 2.6 g/100 g dry weight), the GI of pounded yam (GI 85) was significantly higher than that of attieke. As a matter of fact, the presence of certain food components in attieke such as anti-nutrients including phytic acid, polyphenols, lectins, organic acids, and salts may reduce the rate of starch digestion and affect the postprandial GR [40]. Especially, the organic acids compounds (lactic acids, acetic, propionic acids, *etc.*) could have lowered the GI of attieke, as attieke is a fermented product [20] contrary to pounded yam.

The amount and type of carbohydrate are the main dietary factors affecting insulin secretion and postprandial glycaemia as described by the relationship between the GI values and total dietary fiber content (Spearman’s ρ = −0.407; *p* < 0.01), total content CHO (Spearman’s ρ = −0.286; *p* < 0.01) and available CHO content (Spearman’s ρ = −0.335; *p* < 0.01) of the tests food in this study. GL evaluated in this study allows comparisons of the glycaemic effect of realistic portions of different foods. It has been calculated by multiplying the amount of carbohydrate in one serving by the GI of the food (*i.e.*, GL _placali_ = (GI _placali_ × 15.8)/100 g) [24]. Attieke (GL 29 g (SD 3)/test feed serving), pounded cassava-plantain (GL 26 g (SD 3)/test feed serving) and pounded yam (GL 22 g (SD 4)/test feed serving) have high GLs while maize meal stiff porridge and placali have medium GLs, 16 g (SD 3)/test feed serving and 17 g (SD 3)/test feed serving respectively, according to the GL classification system described by Venn and Green [26]. Although, attieke (GI 63) and placali (GI 106) are both made of cassava with the different GIs, the GL of attieke (GL 29) is significantly higher than placali (GL 17) (*p* < 0.05, Table 3); the case of maize meal stiff porridge (GI 74; GL 16) is similar. The simple reason is that, there is not a lot of carbohydrate in a serving of placali (15.8 g/100 g dry weight *vs*. 46.2 g/100 g dry weight for attieke, *p* < 0.05), since most of it is fiber (8.4 g/100 g dry weight) and water (81.0 g/100 g; Table 2). The relationship between GL and GI is not straightforward; higher-GI foods can have medium GL, and also GL depends upon the portion size eaten [41]. As already noted by Mendosa [41], foods that have a low GL almost always have a low GI, and foods with an intermediate or high GL range from very low to very high GI. Therefore, people who are more concerned about their postprandial glucose response should be very cautious about their serving size of food, for higher-GL foods may raise the blood glucose response. A low-GL diet from either placali or pounded cassava-plantain or maize meal stiff porridge or pounded yam can be achieved by choosing small servings of foods [42]. That is why, on the basis of food consumption per day in respect to GL <80 [41,43], the consumption of placali, pounded cassava-plantain, pounded yam, attieke and maize meal stiff porridge should be limited to 1554 g, 500 g, 751 g, 207 g and 313 g per day respectively regardless of their respective GI in order to avoid metabolic disturbance related to their overconsumption [44].

A limitation of the present study is the use of varying methodology by different laboratories for the measurement and calculation of GI that may contribute to the variation seen in published GI values. We did not undertake measurements of insulin in the present study due to financial constraints but also because our main objective was to generate GI/GL data for Ivorian foods. Besides, the generalization of these conclusions must, however allow for potential variation induced by processing and cooking methods and the possible differences in variety and especially maturity of some of the components of these diets. Acceptability of the meals raised some concerns. Some of the subjects complained about the glucose water (250 mL + 50 g glucose) saying it was too sweet and some about the foods tested. For some subjects, the consumption of test foods without sauces is acceptable, but for other subjects, it is unacceptable. Despite this, all the subjects consumed their glucose water (standard food) and all test foods without any problems.

## 5. Conclusions

The present study has provided reliable values of GI and GL for foods commonly consumed in Côte d’Ivoire. The majority of foods tested have high GI. The consumption of attieke agbodjama could minimize postprandial blood glucose spikes, in spite of high GL and potentially have benefit in the management and prevention of some chronic diseases. The prevalence of diabetes mellitus is still increasing in Côte d’Ivoire and many healthy Ivorian as well as diabetic customers consume the foods studied. Therefore, food processing methods that reduce the GI should be explored. Moreover this study can provide useful guidance for health workers involved in meal planning for diabetics and diabetes education programmes. It should now be possible to advocate for evidence-based changes in the type and frequency of staples used by Ivorian individuals. More studies on GI values for other foods consumed in Côte d’Ivoire need to be conducted and thus to extend the database of metabolic effects of African meals.
